# *Bacillus paralicheniformis* LN33 fermented feed improves growth performance in Cherry Valley ducks by enhancing immune function and intestinal barrier integrity

**DOI:** 10.3389/fvets.2025.1619287

**Published:** 2025-07-23

**Authors:** Yilong Jiang, Xiaofei Yang, Yi Lei, Songlin Li, Xianxin Chen, Li Jiang

**Affiliations:** ^1^Leshan Academy of Agriculture Science, Leshan, Sichuan, China; ^2^College of Life Science and Agri-Forestry, Southwest University of Science and Technology, Mianyang, Sichuan, China

**Keywords:** *Bacillus paralicheniformis* LN33, Cherry Valley ducks, fermented feed, growth performance, immune function, intestinal barrier

## Abstract

**Introduction:**

This study investigated the effects of feed fermented with *Bacillus paralicheniformis* LN33 on growth performance, antioxidant capacity, immune response, intestinal barrier function, and gut microbiota in Cherry Valley ducks.

**Methods:**

A total of 480 healthy 7-day-old Cherry Valley ducks (197.33 ± 5.90 g) were randomly divided into four groups. One group received a basal diet (control), while the other three received the basal diet supplemented with 1%, 3%, or 5% fermented feed for 28 days.

**Results:**

Ducks fed 3% fermented feed showed significantly higher final body weight (3,020.00 ± 52.20 g) and average daily gain (100.79 ± 1.73 g) than the control group (2,896.00 ± 120.93 g and 96.39 ± 4.23 g, respectively; *P* < 0.05). The feed-to-gain ratio decreased significantly (1.79 ± 0.03 vs. 1.87 ± 0.08; *P* < 0.05), with similar feed intake across groups. Antioxidant enzyme activity increased, while pro-inflammatory cytokine levels decreased. Expression of intestinal tight junction proteins and immune markers improved. The relative abundances of *Faecalibacterium, Odoribacter*, and *Butyricicoccus* increased significantly and were positively correlated with intestinal and immune function.

**Discussion:**

These results suggest that *B. paralicheniformis*-fermented feed enhances growth performance and overall health in Cherry Valley ducks by boosting antioxidant defenses, modulating immune responses, and reshaping the gut microbiota.

## 1 Introduction

Antibiotics were once widely employed in animal production as the worldwide livestock and poultry industries expanded rapidly. Initially, antibiotics showed remarkable effects—not only enhancing the growth performance of livestock and poultry and improving feed conversion efficiency but also playing a positive role in preventing various bacterial diseases (1). However, over time, this widespread use began to reveal a series of serious problems. The long-term misuse and overuse of antibiotics have led to the emergence of antibiotic-resistant strains, which can be transmitted to humans through the food chain, posing a threat to public health (2). As a result, many countries have introduced. policies to restrict or ban the use of antibiotics in feed to reduce the risk of antibiotic resistance. For example, the European Union has completely banned the use of antibiotic growth promoters since 2006 (3), and China officially implemented a “feed antibiotic ban” policy in 2020 to promote antibiotic-free and environmentally friendly farming practices (4). Additionally, feed safety issues—such as contamination with mycotoxins, heavy metals, and veterinary drug residues—have raised further concerns, as these factors not only jeopardize animal health but also pose risks to food safety (1). These challenges have intensified the search for functional, natural feed additives that can serve as effective alternatives to antibiotics in livestock and poultry production

Antibiotics were historically used extensively in livestock and poultry production due to their ability to enhance growth performance, improve feed conversion efficiency, and prevent bacterial infections (2). However, the long-term misuse and overreliance on antibiotics have contributed to the rise of antimicrobial resistance, with resistant strains capable of entering the human food chain and posing significant public health risks (3). In light of these concerns, many countries have enacted strict regulations on antibiotic use in animal feed—for example, the European Union banned antibiotic growth promoters in 2006 (4), and China implemented a national feed antibiotic ban in 2020 to promote safer, more sustainable farming practices (5).

Probiotics are considered potential alternatives to antibiotics due to their ability to enhance gut microbial balance, enhance immune function, and promote growth (6). For example, *lactic acid bacteria* can lower intestinal pH by producing metabolites such as acetic acid and lactic acid, thereby inhibiting the growth of pathogenic bacteria (7); *Saccharomyces cerevisiae* can enhance animals' digestive capacity and promote the proliferation of beneficial microbiota (8). Among various probiotics, *Bacillus paralicheniformis* has gained increasing attention in livestock and poultry farming due to its excellent spore-forming ability, secretion of antimicrobial substances, and production of digestive enzymes. *Bacillus paralicheniformis* is a facultative anaerobic, spore-forming probiotic with strong environmental adaptability and metabolic activity (9). It can form heat- and acid-resistant spores in the gastrointestinal tract, improving survival rates. *Bacillus paralicheniformis* has been reported to produce antimicrobial peptides such as bacitracin, which exhibit strong activity against Gram-positive pathogens and remain stable under various environmental conditions (10). Genomic analysis also reveals the presence of multiple biosynthetic gene clusters related to antibacterial activity and environmental adaptation (11), suggesting its potential role in suppressing harmful microorganisms in complex ecological niches. Additionally, *Bacillus paralicheniformis* can produce a variety of digestive enzymes, such as proteases and amylases (12), to promote the breakdown and absorption of nutrients in feed, thereby improving feed utilization and enhancing the growth performance and overall health of livestock and poultry. However, in practical applications, directly adding probiotics to feed still faces several challenges. For instance, probiotics added directly to feed often suffer from poor stability during feed processing and storage, leading to reduced viability and inconsistent efficacy in the gastrointestinal tract, especially under high temperature or humidity conditions, which restricts their practical use in livestock and poultry production (13–16).

In recent years, using probiotics to ferment feed has become an important strategy to enhance the effectiveness of probiotics. Fermented feed not only improves feed digestibility and nutritional value (17) but also produces bioactive metabolites through microbial fermentation, which can help regulate the intestinal microbial community (18) and enhance animals' antioxidant capacity and immune function (19). The fermentation process can reduce anti-nutritional factors in the feed (17) and decrease the incidence of intestinal inflammation (20). Therefore, probiotic-fermented feed holds great potential for application in livestock and poultry farming.

Although probiotic-fermented feed has shown promise in poultry production, most existing studies have focused on broilers or laying hens. In contrast, Cherry Valley ducks, a commercially significant breed known for their fast growth and meat quality, remain underrepresented in probiotic research. Moreover, their distinct gut physiology and microbiota composition may yield different responses to fermented feed interventions. A recent study demonstrated that supplementation with *Bacillus toyonensis* BCT-7112^T^ in Barbary ducks significantly improved growth performance, modulated immune-related gene expression, and reshaped gut microbial composition (21). Although the duck breed and Bacillus strain differ, their work supports the potential of Bacillus-based probiotic interventions in meat-type ducks and highlights the need to evaluate strain- and species-specific effects. To address this gap, we systematically evaluated the effects of *Bacillus paralicheniformis* LN33-fermented feed on growth performance, serum antioxidant capacity, immune function, intestinal barrier integrity, and gut microbial composition in Cherry Valley ducks. This study not only provides new insight into the use of LN33 as a functional probiotic strain but also helps establish a scientific basis for optimizing duck production using microbiota-targeted nutritional strategies.

## 2 Materials and methods

### 2.1 Experimental design and preparation of fermented feed

Table 1 displays the basal diet that was employed in this trial. It was created in compliance with NY/T 2122–2012, the feeding guideline for meat ducks. Table 2 lists the ingredients that make up the fermented feed. *Bacillus paralicheniformis* LN33, which has been kept at the China General Microbiological Culture Collection Center (CGMCC) with accession number CGMCC No. 24305, was isolated from the cecal contents of laying hens. Preparation of fermentation broth: *Bacillus paralicheniformis* LN33 preserved in glycerol was inoculated into LB liquid medium and cultured at 37°C with shaking at 180 r/min for 24 h to prepare the seed culture. The seed culture was then inoculated into an expansion liquid medium at a 1% inoculation rate and cultured for another 24 h to produce the fermentation broth. Preparation of fermented feed: in short, the fermentation ingredients listed in Table 2 were weighed according to their respective proportions and mixed thoroughly in a mixer. The mixture was then packed into feed fermentation bags equipped with one-way air valves and fermented under anaerobic conditions at 35°C for 96 h. The fermented feed was used on the day fermentation was completed to avoid post-fermentation spoilage. The viable *Bacillus paralicheniformis* count was confirmed to be at least 1 × 10^6^ CFU/g in each batch to ensure effective live bacterial delivery. During feeding, it was mixed thoroughly with pelleted feed according to the designated group ratios to ensure uniform intake.

**Table 1:** Dietary formula composition and nutritional levels.

**Raw material (%)**	**1–14 days**	**15–36 days**	**Nutritional level**	**1–14 days**	**15–36 days**
Corn	50.00	48.50	AME (MJ/kg)	12.18	12.55
Soybean meal	24.00	14.00	CP (%)	19.79	17.44
Wheat middlings	7.50	14.65	Ca (%)	0.97	0.91
Rice bran	6.40	7.30	AP (%)	0.42	0.38
Corn gluten meal	5.00	8.00	Lys (%)	0.99	0.80
Soybean oil	2.00	2.50	Met (%)	0.45	0.44
CaCO_3_	1.50	1.40	Trp (%)	0.22	0.19
CaHPO_4_	1.50	1.50	Thr (%)	0.74	0.66
NaCl	0.37	0.37	Met + Cys (%)	0.85	0.81
L-Lysine HCl (98%)	0.15	0.20			
DL-Met (99%)	0.15	0.15			
Premix	1.00	1.00			
Palygorskite	0.43	0.43			
Total	100.00	100.00			

The premix provided the following per kilogram of the basal diet: V_A_ 10,000 IU, V_D3_ 2,000 IU, V_E_ 20.0 mg, V_K_ 0.5 mg, V_B1_ 3.0 mg, V_B2_ 4.0 mg, V_B3_ 60.0 mg, calcium pantothenate 11.0 mg, V_B6_ 2.5 mg, V_B7_ 0.2 mg, V_B9_ 0.6 mg, V_B12_ 0.04 mg, CuSO_4_·5H_2_O 8.0 mg, FeSO_4_·7H_2_O 80.0 mg, ZnSO_4_·H_2_O 60.0 mg, Na_2_SeO_3_ 0.2 mg, KI 0.4 mg, MnSO_4_·H_2_O 80.0 mg.

**Table 2:** Composition of fermented feed ingredients.

**Raw material**	**Composition (%)**
Corn	20
Soybean meal	15
Wheat bran	24
Glutamic acid residue	17
Fermentation broth	8
Water	16
Total	100
Nutritional level	
Metabolizable energy (MJ/kg)	12.13
Crude protein (%)	17.46
Calcium (%)	0.65
Phosphorus (%)	0.33
Lysine (%)	0.71
Methionine (%)	0.41

### 2.2 Experimental ducks and feeding management

This experiment was carried out at a Leshan City meat duck farm. Four food groups (CON, FF1, FF2, and FF3) were randomly assigned to 480 healthy 7-day-old Cherry Valley ducks, each weighing an average of 197.33 ± 5.90 g. Each group had six replicates, with 20 ducks per replication. While the FF1, FF2, and FF3 groups received 1%, 3%, and 5% fermented feed supplements, respectively, the CON group was given a baseline diet. The ducks were kept in elevated wire cages, with 20 ducks per cage (cage dimensions: 2 m × 1 m × 0.75 m). Each cage was equipped with a feed trough and nipple drinkers, allowing *ad libitum* access to feed and water. Prior to the trial, the housing area was thoroughly cleaned and disinfected. A 1% sodium hydroxide solution was sprayed for general disinfection, while feed buckets, troughs, and drinkers were disinfected with a 2% peracetic acid solution. The facility was sealed for more than 2 h, followed by ventilation for 3 days before the trial began. Since ducklings have short down feathers and underdeveloped thermoregulation, appropriate environmental temperatures were maintained. Before the ducklings were placed, the room temperature was raised to 30–33°C For the first 3 days after placement, this temperature was maintained. From days 4 to 7, the temperature was kept at 27–29°C and thereafter it was reduced by 2–3°C each week until stabilized at ~20°C. The relative humidity of the housing was maintained at 60%−70%, with continuous 24-h lighting. Disease prevention and hygiene management were carried out according to standard farm protocols. Ducks were fed four times daily—at 8:00, 12:00, 16:00, and 20:00. The feeding method involved using pelleted feed mixed with fermented feed to meet the ducks' nutritional needs and improve feed utilization. The fermented feed, prepared according to the Leshan Anyou fermented feed formula, was mixed into the pelleted feed at the designated ratios and fed after thorough mixing. Each feeding portion was adjusted to leave a small amount of residual feed in the troughs. The fermented feed, prepared according to the Leshan Anyou fermented feed formula, was mixed into the pelleted feed at the designated ratios and fed after thorough mixing. Each feeding portion was adjusted to leave a small amount of residual feed in the troughs. The fermented feed, prepared according to the Leshan Anyou fermented feed formula, was mixed into the pelleted feed at the designated ratios and fed after thorough mixing. Each feeding portion was adjusted to leave a small amount of residual feed in the troughs. The fermented feed was used on the day of fermentation completion to prevent secondary fermentation or spoilage. To ensure consistency, each batch of fermented feed was freshly prepared following the specified fermentation time, and nutrient content was analyzed to confirm quality before feeding. The fermented feed was freshly prepared for each batch and used on the day of fermentation completion to ensure consistency and prevent secondary fermentation or spoilage. All batches were freshly prepared following the same fermentation duration and conditions described in the protocol. Nutrient content was analyzed for each batch to ensure consistent microbial activity and composition across treatments. Feed troughs and drinkers were cleaned daily to prevent contamination. During feeding, the health condition of the ducks was monitored. Ducks showing signs of lethargy or reduced appetite were closely observed. If no improvement was seen over time, they were isolated and fed separately, with proper records kept. The trial lasted for 28 days. The experimental design is shown in Table 3.

**Table 3:** Experimental design (Total *n* = 480).

**Group**	**Treatment**	**Replicates**	**Ducks per cage**	**Ducks per group**
CON	Basal diet	6	20	120
FF1	Basal diet + 1% fermented feed	6	20	120
FF2	Basal diet + 3% fermented feed	6	20	120
FF3	Basal diet + 5% fermented feed	6	20	120

### 2.3 Sample collection

At the end of the feeding trial, after a 12-h fasting period, one healthy meat duck with a body weight close to the average was selected from each replicate. Approximately 5 ml of blood was collected from the wing vein for the determination of serum immune parameters and antioxidant enzyme activity. After blood collection, the duck was euthanized using carbon dioxide. Once complete unconsciousness was confirmed, the duck was exsanguinated and immediately dissected. Adipose tissue and any associated fascia were meticulously clipped after the heart, liver, pancreas, gizzard, spleen, thymus, and bursa of Fabricius were quickly removed. Using sterile scissors, a 2- to 3-cm piece from the middle of the duodenum, jejunum, and ileum was cut. The intestinal contents were then rinsed out with saline and preserved in 50 ml of 4% paraformaldehyde solution for histological examination. A 10-cm slice from the middle of the ileum, jejunum, and duodenum was also cut open. A sterile slide was used to scrape off the mucosa after the intestinal contents were gently washed away with saline. After being wrapped in foil, tagged, and snap-frozen in liquid nitrogen, the mucosal samples were put into 5-ml cryogenic tubes and kept at −80°C for further examination. Under anaerobic conditions, sterile scissors were used to open the middle region of the cecum, and the contents were squeezed out into sterile 5-ml cryogenic tubes. These samples were snap-frozen in liquid nitrogen and kept at −80°C for future use.

### 2.4 Index measurements

#### 2.4.1 Measurement of production performance

Using each replicate as a unit, the ducks were weighed after fasting for 12 h at 8:00 a.m. on days 1, 14, and 35 of the trial. Feed intake, residual feed, and wasted feed were recorded for each replicate. The body weight of ducks in each replicate was also recorded. These data were used to calculate average daily feed intake (ADFI), average daily gain (ADG), and feed-to-gain ratio (F/G).


$$\begin{array}{c} \text{ADFI }=\;\left(\text{Feed offered}-\text{Residual feed}-\text{Wasted feed}\right)/\\ \;\left(\text{Number of trial days }\times \text{ Number of ducks}\right)\\ \text{ADG }=\;\left(\text{Final body weight}-\text{Initial body weight}\right)/\\ \;\left(\text{Number of trial days }\times \text{ Number of ducks}\right)\\ \text{F}/\text{G }=\text{ ADFI}/\text{ADG} \end{array}$$

#### 2.4.2 Slaughter performance and organ index measurement

At the end of the feeding trial, one healthy meat duck close to the average body weight was selected from each replicate. The live weight was measured using an electronic scale and recorded. Subsequently, blood was collected via the wing vein after disinfecting the blood collection site, and a sufficient amount of blood was drawn using a syringe for serum antioxidant and immunoglobulin measurements. After blood collection, the duck was euthanized using carbon dioxide. Once it lost consciousness, exsanguination was performed. After removing the feathers, foot keratin layer, toenails, and beak, the carcass weight was recorded. The carcass was then further processed by removing the trachea, esophagus, crop, intestines, spleen, pancreas, gallbladder, reproductive organs, gizzard contents, and keratin membranes, and the semi-eviscerated weight was recorded. Next, the heart, liver, proventriculus, gizzard, lungs, and abdominal fat were removed, and the full eviscerated weight was recorded. After removing the skin and bones from the duck's legs, the leg muscle weight was recorded. The breast muscle was completely separated, and the breast muscle weight was recorded. The following indices were calculated:


$$\begin{array}{l} \text{ Slaughter yield }=\;(\text{Carcass weight}/\text{Live weight})\\ \;\times \;100\%\\ \text{Semi }-\text{ eviscerated yield }=\;(\text{Semi}-\text{eviscerated weight}/\\ \text{ Live weight})\;\times \;100\%\\ \text{ Full eviscerated yield }=\;(\text{Full eviscerated weight}/\text{Live weight})\\ \;\times \;100\%\\ \text{ Leg muscle yield }=\;(\text{Leg muscle weight}/\text{Full eviscerated }\\ \text{ weight})\;\times \;100\%\\ \text{ Breast muscle yield ​​​​}(\text{Breast muscle weight}/\text{Full eviscerated }\\ \text{ weight})\times \;100\% \end{array}$$

After dissection, the heart, liver, pancreas, gizzard, spleen, thymus, and bursa of Fabricius were quickly removed, and any attached fascia and adipose tissue were trimmed. Surface moisture was absorbed using absorbent paper, and the organs were weighed and recorded to calculate the organ index.


$$\begin{array}{c} \text{Organ index }=\;\left[\text{Organ weight }\left(\text{g}\right)/\text{Live weight }\left(\text{kg}\right)\right]\;\times \;100\%\; \end{array}$$

#### 2.4.3 Measurement of serum antioxidants and immunoglobulins

Following blood collection, the blood was placed in red-cap vacuum blood collection tubes devoid of anticoagulant and left to stand for half an hour at room temperature. After that, the samples were centrifuged for 10 min at 4°C at 3,500 rpm. For later usage, the top serum was carefully moved into 1.5 ml sterile centrifuge tubes and kept at −20°C Serum levels of immunoglobulin A, immunoglobulin G, and immunoglobulin M were measured using the enzyme-linked immunosorbent assay (ELISA). A microplate reader or spectrophotometer was used to measure the following: total antioxidant capacity, malondialdehyde, superoxide dismutase, catalase, glutathione peroxidase, interleukin-1 beta, interleukin-2, interleukin-6, interferon-gamma, tumor necrosis factor-alpha, and interleukin-10.

#### 2.4.4 Measurement of intestinal morphology

Using sterile scissors, 2–3 cm segments of the duodenum, jejunum, and ileum were collected. The intestinal contents were washed with physiological saline and then fixed in 50 ml of 4% paraformaldehyde solution for histological analysis. The intestinal tissue morphology was assessed following tissue embedding, hematoxylin-eosin (HE) staining, and slide examination. The specific steps are as follows: the three intestinal segments were processed for paraffin embedding, which involved tissue trimming, dehydration, clearing, wax infiltration, and embedding. Thin sections of 5 μm were cut, followed by deparaffinization, rehydration, and hematoxylin-eosin (HE) staining. After staining, the sections were dehydrated again, cleared, and mounted using neutral balsam. The slides were then observed under a microscope and images were captured. The villus height and crypt depth were measured using ImageJ software, and the villus-to-crypt ratio was calculated.

#### 2.4.5 Gene expression analysis of jejunal mucosa

The TRIzol technique was used to extract total RNA from the jejunal mucosa. Total RNA was extracted using the TRIzol reagent (15596026CN, Thermo) in accordance with the manufacturer's instructions. Total RNA quality and concentration were assessed using 1% agarose gel electrophoresis and a protein-nucleic acid spectrophotometer (ND-2000 UV, Thermo Fisher, USA). If a sample's OD260/280 ratio was between 1.8 and 2.1, it was considered acceptable. cDNA synthesis was performed using a reverse transcription kit (RR047A, Takara) in accordance with the reaction system and parameters listed in the kit's instructions. The produced cDNA was used as a template for fluorescence quantitative PCR amplification using a fluorescence quantitative PCR kit (RR820A, Takara) in accordance with the manufacturer's instructions. Using a CFX96 apparatus (Bio-Rad, USA), the fluorescence quantitative PCR was carried out. It comprised 5 μl SYBR Green premix, 0.4 μl upstream and downstream primers, 1 μl cDNA template, and 3.2 μl dH_2_O, with a total reaction volume of 10 μl. The amplification program was as follows: a melt curve from 65 to 95°C was produced following a 30-s pre-denaturation at 95°C, 39 cycles of 95°C for 5 s (denaturation), and 60°C for 40 s (annealing/extension) in order to verify the specificity of the amplified products. To assure the accuracy and reproducibility of the experiment, thorough quality control techniques were followed. A no-template control was added in every sample to check for contamination. By serially diluting the cDNA template in a 10-fold gradient and creating a standard curve, the PCR amplification efficiency was ascertained. To determine the amplification efficiency (*E*), the formula *E* = (10 ^(−1/*slope*)^ – 1) × 100% was used. For further analysis, genes with a correlation coefficient (*R*^2^) > 0.99 and an efficiency ranging from 90 to 105% were considered. Beijing Qingke Biotechnology Co., Ltd. produced all of the primers, while GAPDH served as the internal control. The 2^−ΔΔCt^ technique was used to evaluate the relative expression levels of target genes (22). Table 4 presents the primer sequences.

**Table 4:** Real-time quantitative PCR primer sequences.

**Genes name**	**Primer sequence (5** ^**′**^ **-3** ^**′**^ **)**		**Product Size (bp)**	**GenBank acession number**
*β-actin*	F	AGAAATTGTGCGTGACATCAA	227	XM_13108556.1
	R	GGACTCCATACCCAAGAAAGAT		
*ZO-1*	F	ACGCTGGTGAAATCAAGGAAGAA	255	XM_013093747.1
	R	AGGGACATTCAACAGCGTGGC		
*ZO-2*	F	ACAGTGAAAGAAGCTGGCGTAG	131	XM_005019888.2
	R	GCTGTATTCCCTGCTACGGTC		
*CLDN-1*	F	TCCATGCATGTGCTGTTGGC	145	NW_004678814.1
	R	CCTGCTGCAGTTGCAGTGTT		
*CLDN-2*	F	CTCCTCCTTGTTCACCCTCATC	160	XM_005009661.2
	R	GAACTCGCTCTTGGGTTTGTG		
*OCLN*	F	CAGGATGTGGCAGAGGAATACAA	160	XM_013109403.1
	R	CCTTGTCGTAGTCGCTCACCAT		
*MUC-2*	F	GGGCGCTCAATTCAACATAAGTA	150	XM_005024513.2
	R	TAAACTGATGGCTTCTTATGCGG		
*sIgA*	F	TCGCTCAAGGAACCCATCGT	174	U27222.1
	R	GCGGGACCACGAGAACTTCA		
*TNF-α*	F	ACAGGACAGCCTATGCCAAC	165	XM_005019359.2
	R	ACAGGAAGGGCAACACATCT		
*IL-6*	F	CTGCGAGAACAGCATGGAGA	191	XM_013100522.2
	R	GAAAGGTGAAAAGCCCGCTG		
*IL-10*	F	GCTGGAGATGATGCGGTTCT	179	XM_013092231.1
	R	ATGTCAAACTCCCCCATCGC		

#### 2.4.6 Cecal microbial indicators and gut microbiome metabolomics measurement (cecal content 16S rRNA gene sequencing)

Following the manufacturer's instructions, microbial genomic DNA was extracted from 200 mg cecal content samples of each group using the MagPure Soil DNA LQ Kit (Guangdong Magen, China). The extracted DNA was subjected to concentration and purity testing before being used as the template for subsequent PCR amplification. The bacterial 16S rRNA gene V3–V4 hypervariable region was amplified using Tks Gflex DNA Polymerase (Takara, R060B) and universal primers 343F (5′-TACGGRAGGCAGCAG-3′) and 798R (5′-AGGGTATCTAATCCT-3′) in a 30 μl reaction mixture. After purification, the amplification products were sequenced using the Illumina NovaSeq 6000 high-throughput sequencing platform with paired-end (PE) sequencing, with a read length of 250 bp for each direction (PE250). Sequencing of the 16S rRNA gene amplicons and bioinformatics analysis were performed by Shanghai OE Biotech Co., Ltd. (Shanghai, China).

### 2.5 Statistical analysis

The experimental data were analyzed using SPSS 27.0. Quantitative results are presented as mean ± standard deviation. Data normality was assessed using the Shapiro–Wilk test, and homogeneity of variance was evaluated using Levene's test. When both assumptions were met, differences among groups were analyzed using one-way analysis of variance (ANOVA), followed by Tukey's multiple comparison test as a *post-hoc* analysis. When data did not conform to a normal distribution or equal variance, the Kruskal–Wallis non-parametric test was used instead. A value of *P* < 0.05 was considered statistically significant. All graphs were generated using GraphPad Prism 10.1.2.

## 3 Results

### 3.1 Effect of fermented feed on the growth performance of meat ducks

As shown in Table 5, compared to the CON group, the final body weight and average daily gain of the FF1 and FF2 groups were significantly increased (*P* < 0.05), while the feed-to-gain ratio was significantly reduced (*P* < 0.05). There were no significant changes in the final body weight, ADG, or F/G ratio in the FF3 group compared to the control group (*P* > 0.05). There were no significant differences in the initial body weight and average daily feed intake among the groups (*P* > 0.05).

**Table 5:** Effect of fermented feed on the growth performance of meat ducks.

**Item**	**Group**			
	**CON**	**FF1**	**FF2**	**FF3**
Initial weight (g)	197.13 ± 5.25	197.42 ± 8.38	197.75 ± 5.01	197.04 ± 6.07
Final weight (g)	2,896.00 ± 120.93^b^	3,004.33 ± 70.03^a^	3,020.00 ± 52.20^a^	2,986.50 ± 51.18^ab^
Average daily gain (ADG)	96.39 ± 4.23^b^	100.25 ± 2.74^a^	100.79 ± 1.73^a^	99.62 ± 1.77^ab^
Average daily feed intake (ADFI)	180.27 ± 0.83	179.86 ± 0.54	180.10 ± 0.19	179.88 ± 0.30
Feed-to-gain ratio (F/G)	1.87 ± 0.08^a^	1.80 ± 0.05^b^	1.79 ± 0.03^b^	1.81 ± 0.03^ab^

Different superscript letters in the same row indicate significant differences (P < 0.05), while the same letters indicate no significant difference (P > 0.05).

### 3.2 Effect of fermented feed on slaughter performance of meat ducks

As shown in Table 6, compared to the CON group, the chest muscle rate in the FF1 and FF3 groups was significantly increased (*P* < 0.05). There were no significant changes in slaughter rate, half-eviscerated rate, full-eviscerated rate, and leg muscle rate among the groups (*P* > 0.05). Additionally, there were no significant differences in the organ indices of the heart, liver, spleen, pancreas, glandular stomach, and muscular stomach among the groups (*P* > 0.05).

**Table 6:** Effects of fermented feed on slaughter performance of meat ducks.

**Item**	**Group**			
	**CON**	**FF1**	**FF2**	**FF3**
Slaughter rate	89.28 ± 3.69	88.55 ± 0.91	88.59 ± 0.64	88.27 ± 1.30
Semi-eviscerated rate	82.63 ± 2.26	82.01 ± 0.80	81.29 ± 0.88	81.77 ± 1.75
Full eviscerated rate	74.97 ± 1.98	75.51 ± 1.19	74.86 ± 1.43	75.68 ± 1.70
Leg muscle rate	6.83 ± 0.89	6.63 ± 0.50	6.50 ± 0.89	6.38 ± 0.53
Breast muscle rate	5.92 ± 0.91^b^	7.10 ± 0.72^a^	6.78 ± 0.97^ab^	7.19 ± 0.62^a^
Heart	0.53 ± 0.06	0.52 ± 0.04	0.53 ± 0.02	0.54 ± 0.05
Liver	3.65 ± 0.73	3.31 ± 0.48	3.16 ± 0.49	3.68 ± 0.36
Spleen	0.08 ± 0.01	0.09 ± 0.06	0.09 ± 0.05	0.06 ± 0.01
Pancreas	0.32 ± 0.07	0.33 ± 0.04	0.36 ± 0.04	0.38 ± 0.08
Proventriculus	0.33 ± 0.10	0.26 ± 0.03	0.27 ± 0.02	0.28 ± 0.02
Gizzard	2.13 ± 0.26	2.41 ± 0.22	2.37 ± 0.36	2.23 ± 0.29

Different superscript letters in the same row indicate significant differences (P < 0.05), while the same letters indicate no significant difference (P > 0.05).

### 3.3 Effects of fermented feed on serum antioxidant capacity and immunity

**Figure 1:**
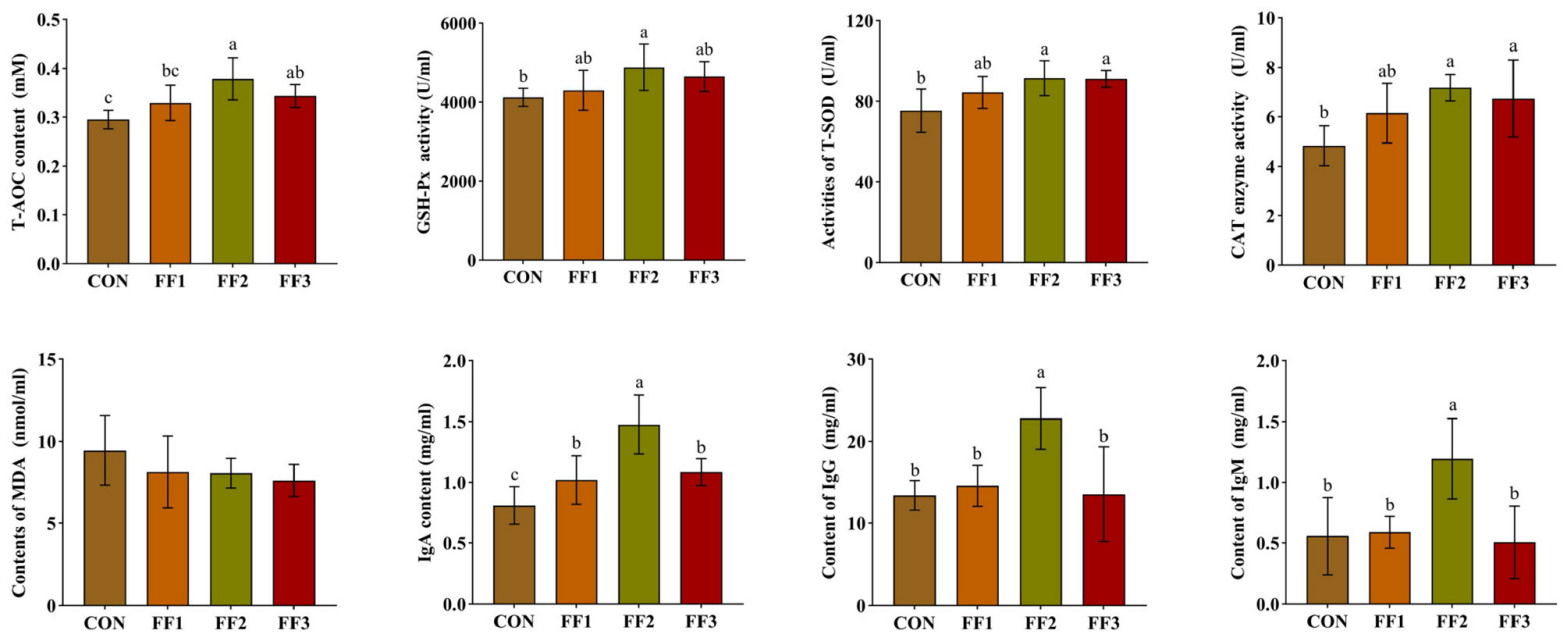
Effects of fermented feed on serum antioxidant capacity and immunoglobulin levels. Different letters indicate significant differences (*P* < 0.05).

As shown in Figure 1, compared with the CON group, the IgA levels in the FF1, FF2, and FF3 groups were significantly increased (*P* < 0.05). The FF2 group showed significantly higher levels of T-AOC, GSH-Px activity, T-SOD activity, CAT activity, IgG, and IgM (*P* < 0.05). The FF3 group showed significantly increased levels of T-AOC, T-SOD activity, and CAT activity (*P* < 0.05). Compared with the CON group, there were no significant changes in T-AOC, GSH-Px activity, T-SOD activity, CAT activity, IgG, and IgM levels in the FF1 group (*P* > 0.05), and no significant changes in GSH-Px activity, IgG, and IgM levels in the FF3 group (*P* > 0.05). Meanwhile, MDA levels showed no significant differences among the groups (*P* > 0.05).

**Figure 2:**
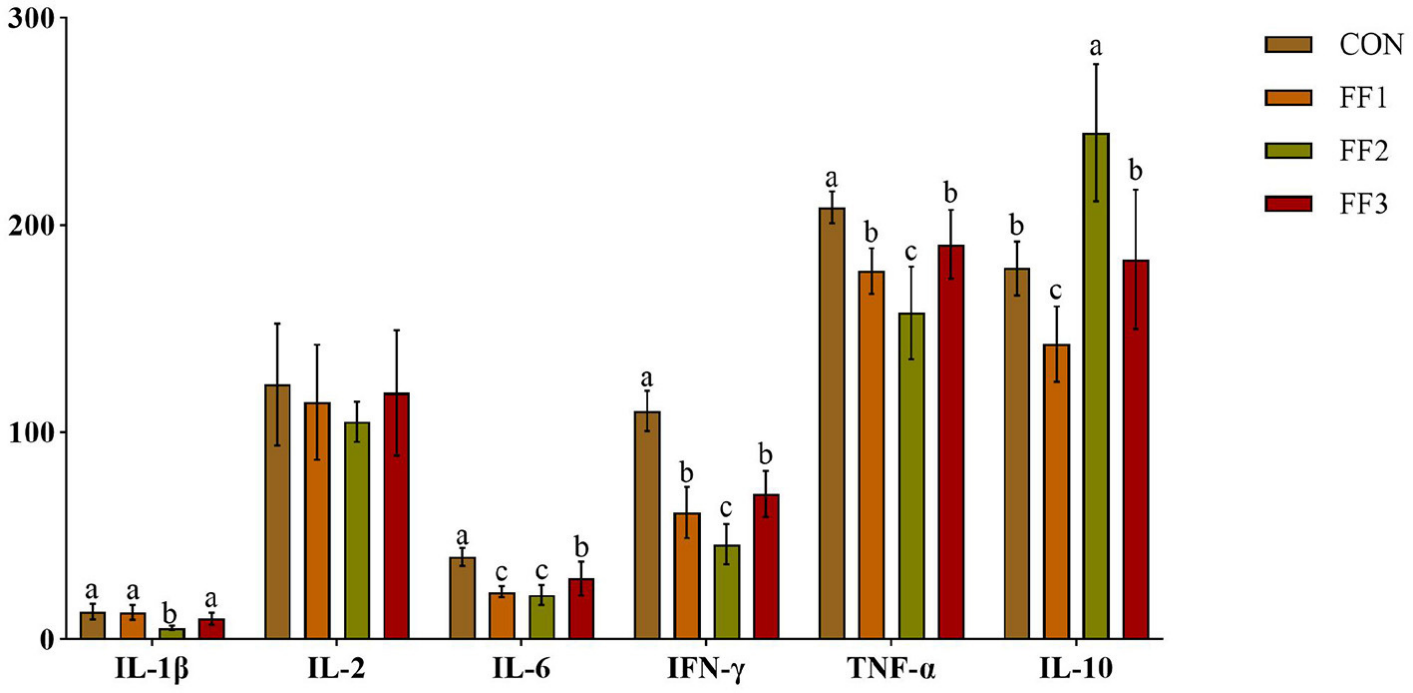
Effects of fermented feed on serum inflammatory factors. Different letters indicate significant differences (*P* < 0.05).

As shown in Figure 2, compared with the CON group, the relative expression levels of IL-6, IFN-γ, and TNF-α in the FF1, FF2, and FF3 groups were significantly decreased (*P* < 0.05). The FF2 group showed a significantly lower relative expression of IL-1β (*P* < 0.05) and a significantly higher relative expression of IL-10 (*P* < 0.05). In contrast, the FF1 group showed a significantly decreased relative expression of IL-10 (*P* < 0.05). Additionally, there were no significant differences in the relative expression levels of IL-2 among the FF1, FF2, and FF3 groups compared to the CON group (*P* > 0.05).

### 3.4 Effects of fermented feed on intestinal morphology in meat ducks

**Figure 3:**
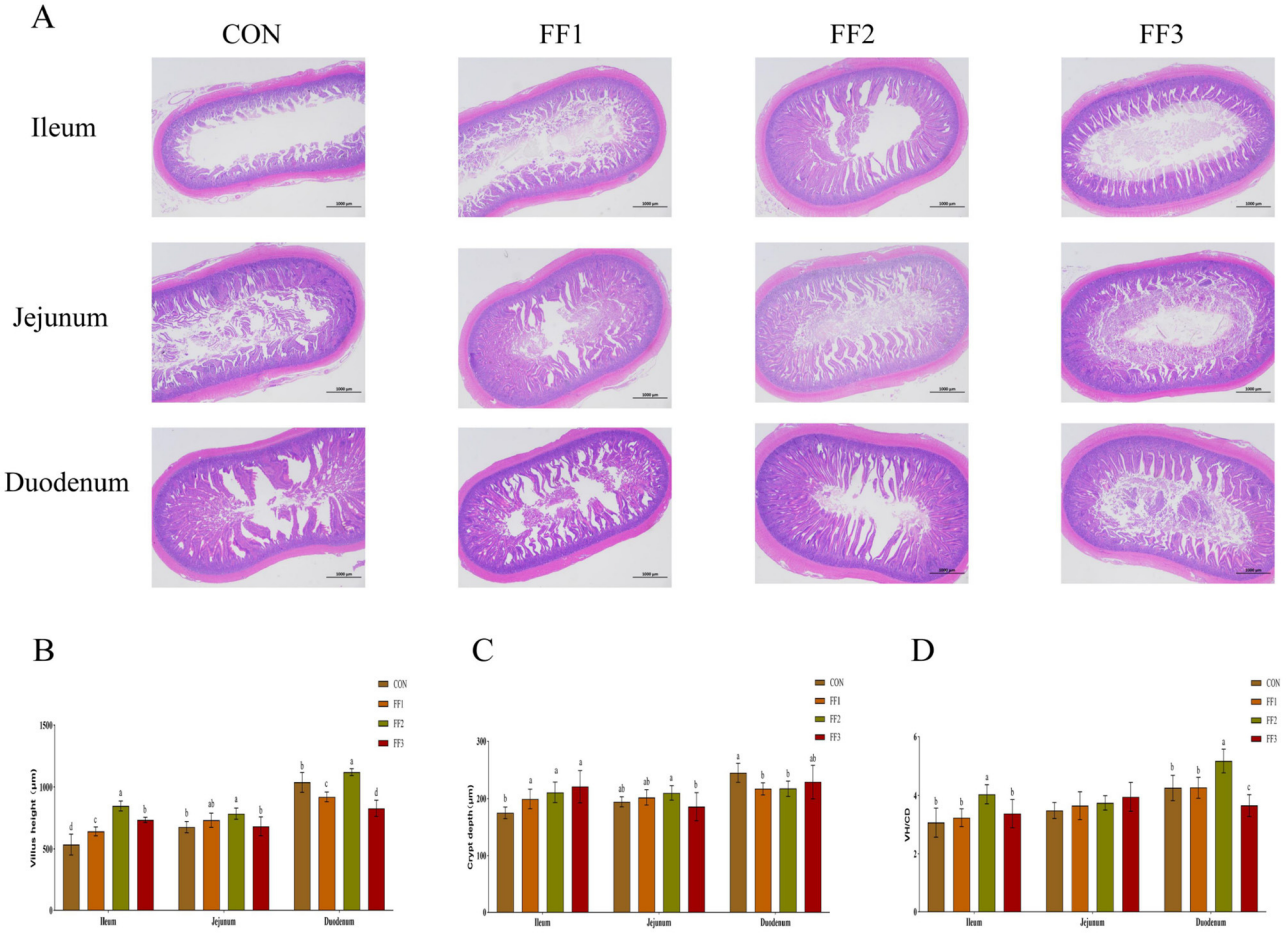
Effects of fermented feed on intestinal morphology. **(A)** Hematoxylin and eosin (HE) staining images of the ileum, jejunum, and duodenum. **(B)** Villus height in the ileum, jejunum, and duodenum. **(C)** Crypt depth in the ileum, jejunum, and duodenum. **(D)** Villus height to crypt depth ratio (VH/CD) in the ileum, jejunum, and duodenum. Different letters indicate significant differences (*P* < 0.05).

As shown in Figures 3A, B, compared with the CON group, the intestinal villi in the ileum of the FF1, FF2, and FF3 groups were significantly increased (*P* < 0.05). The intestinal villi in the jejunum of the FF2 group were significantly increased (*P* < 0.05), while there were no significant changes in the jejunal villi of the FF1 and FF3 groups (*P* > 0.05). For the duodenum, compared with the CON group, the villi in the FF2 group were significantly increased (*P* < 0.05), while the villi in the FF1 and FF3 groups were significantly reduced (*P* < 0.05). As shown in Figure 3C, compared with the CON group, the crypt depth in the ileum of the FF1, FF2, and FF3 groups was significantly increased (*P* < 0.05). The crypt depth in the duodenum of the FF1 and FF2 groups was significantly decreased (*P* < 0.05). For the jejunum, no significant changes in crypt depth were observed in the FF1, FF2, and FF3 groups compared to the CON group (*P* > 0.05). Additionally, no significant changes were observed in the crypt depth of the duodenum in the FF3 group compared to the CON group (*P* > 0.05). As shown in Figure 3D, regarding the ratio of villus height to crypt depth, the FF2 group showed a significant increase in the ratio in the ileum and duodenum compared to the CON group (*P* < 0.05), while the FF3 group showed a significant decrease in the ratio in the duodenum (*P* < 0.05). Compared to the CON group, there were no significant changes in the ratio in the ileum for the FF1 and FF3 groups (*P* > 0.05), nor were there any significant changes in the ratio in the jejunum for the FF1, FF2, and FF3 groups (*P* > 0.05). Additionally, no significant changes were observed in the ratio of the duodenum in the FF1 group (*P* > 0.05).

### 3.5 Effects of fermented feed on intestinal gene expression

**Figure 4:**
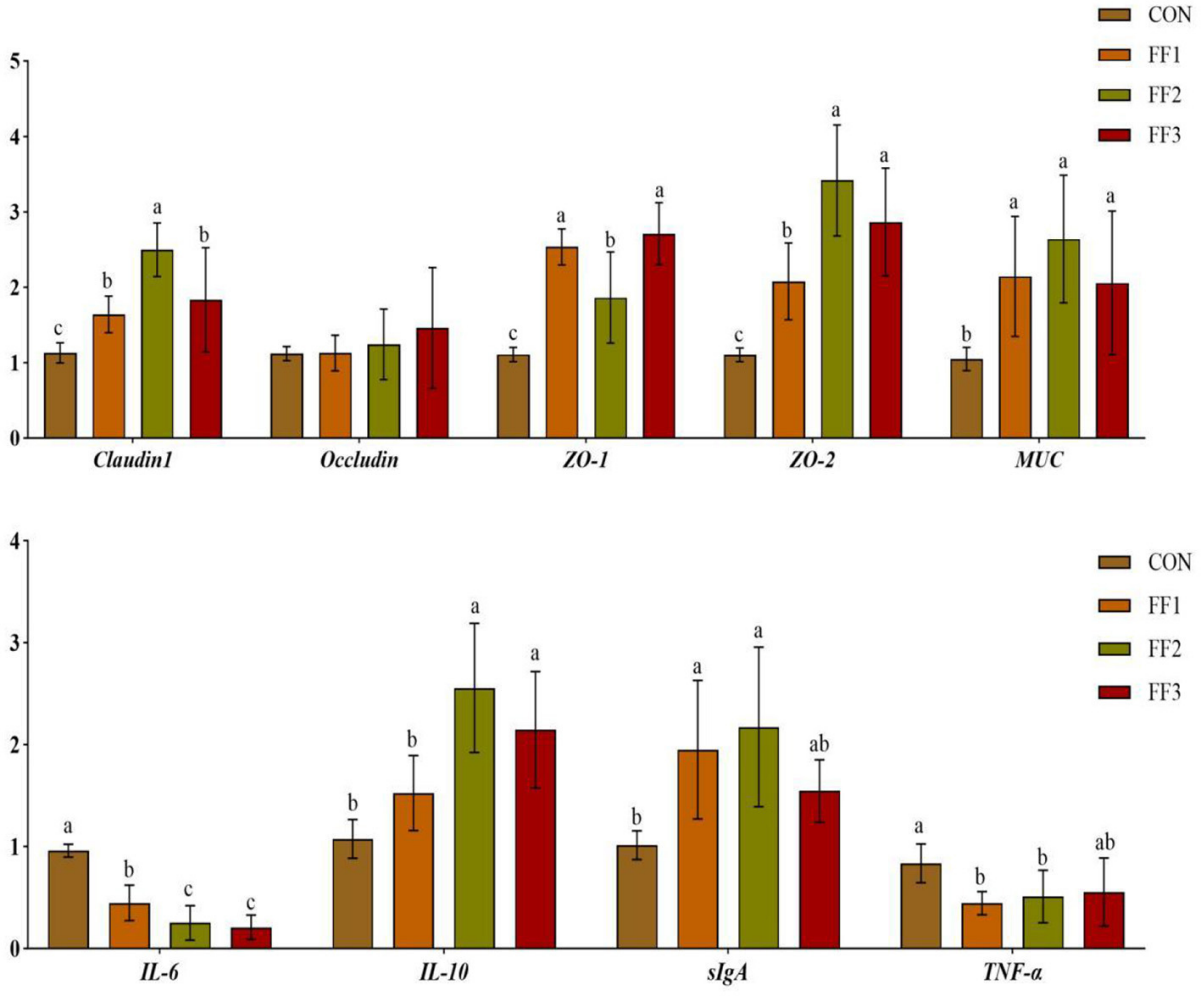
Effects of fermented feed on intestinal gene expression. Different letters indicate significant differences (*P* < 0.05).

As shown in Figure 4, compared with the CON group, the relative mRNA expression levels of *Claudin1, ZO-1, ZO-2*, and *MUC* were significantly increased in the FF1, FF2, and FF3 groups (*P* < 0.05), while the mRNA expression of *IL-6* was significantly decreased (*P* < 0.05). Compared with the CON group, the mRNA expression of *IL-10* was significantly increased in the FF2 and FF3 groups (*P* < 0.05), the expression of *sIgA* was significantly increased in the FF1 and FF2 groups (*P* < 0.05), and the expression of *TNF-*α was significantly decreased in the FF1 and FF2 groups (*P* < 0.05). There were no significant differences in *IL-10* mRNA expression in the FF1 group compared with the CON group (*P* > 0.05), and no significant differences in *sIgA* and *TNF-*α mRNA expression in the FF3 group (*P* > 0.05). There were also no significant differences in *Occludin* mRNA expression among all groups (*P* > 0.05).

### 3.6 Effects of fermented feed on cecal microbiota in meat ducks

**Figure 5:**
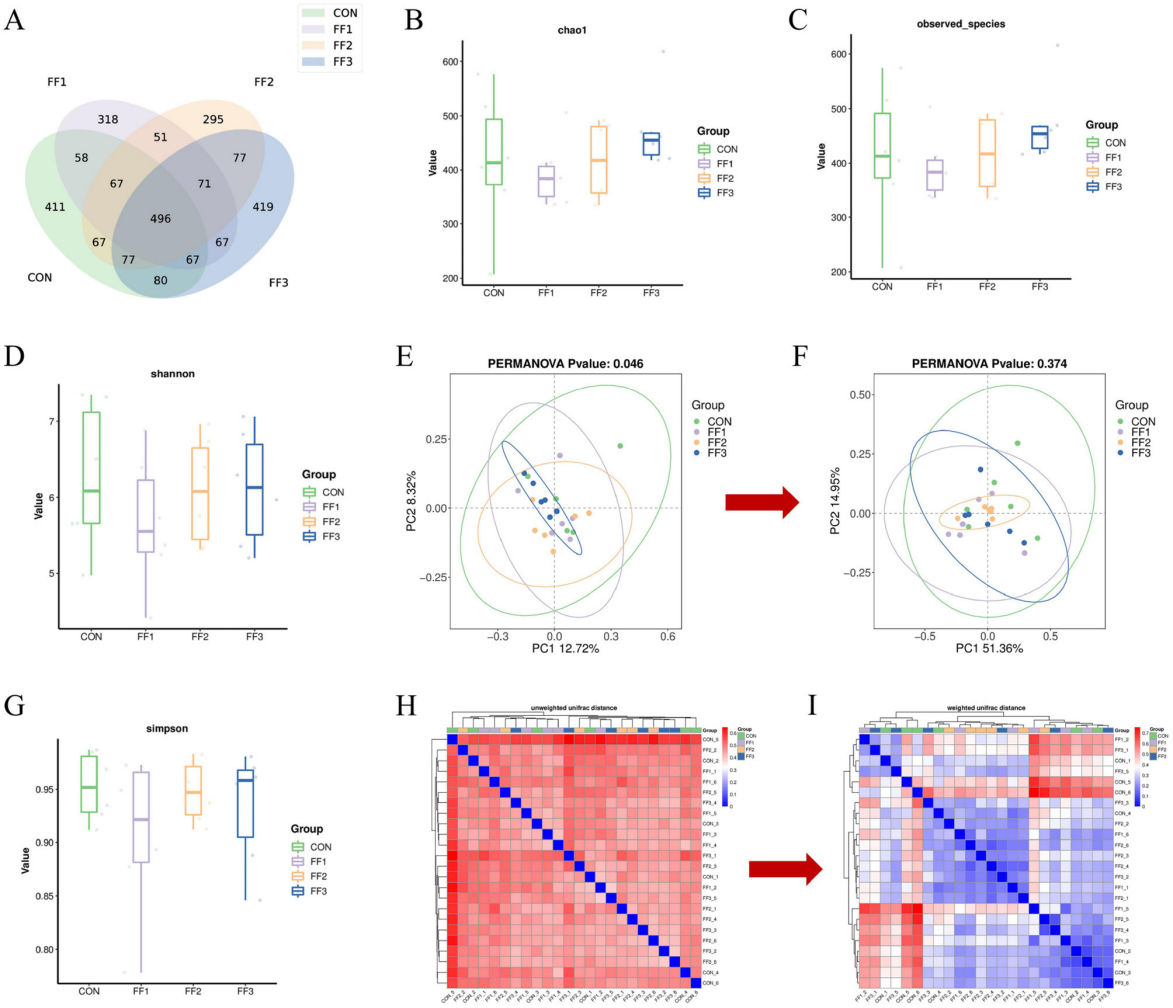
Effects of fermented feed on intestinal microbiota in meat ducks. **(A)** ASV Venn graph. **(B)** Chao1 index. **(C)** Observed species index. **(D)** Shannon index. **(E)** Unweighted UniFrac PCoA analysis. **(F)** Weighted UniFrac PCoA analysis. **(G)** Simpson index. **(H)** Unweighted UniFrac Distance Beta Diversity Analysis. **(I)** Weighted UniFrac Distance Beta Diversity Analysis. Color intensity represents sample similarity (blue: higher similarity; red: lower similarity).

To assess the impact of fermented feed on the intestinal microbiota of meat ducks, 16S rRNA gene sequencing was performed on cecal content samples. As shown in Figure 5A, there were 411 unique ASVs in the CON group, 318 in the FF1 group, 295 in the FF2 group, and 419 in the FF3 group, with 496 ASVs shared across all four groups. Figures 5B–D, G show no significant differences among the CON, FF1, FF2, and FF3 groups in terms of Chao1, Observed Species, Shannon, and Simpson indices (*P* > 0.05). Figure 5E illustrates that PC1 and PC2 explained 12.72 and 8.32% of the community variation, respectively, accounting for a total of 21.04% of the variance. Different colors represent different experimental groups: the FF3 group (blue) exhibited tight clustering, indicating a more stable microbial structure, while the CON (green) and FF1 (purple) groups showed greater dispersion and individual variability. The FF2 group (orange) displayed a more even distribution, with partial overlap with other groups. The PERMANOVA result (*P* = 0.046) indicated a significant difference in microbial composition among groups (*P* < 0.05), suggesting that fermented feed (FF1, FF2, and FF3) significantly influenced the intestinal microbiota structure. Among these, the FF3 group appeared to have formed a more stable microbial pattern, while the CON and FF1 groups had higher diversity but more dispersed structures. The FF2 group lay between these two in terms of stability. Unlike Unweighted UniFrac, Weighted UniFrac incorporates an abundance information. As shown in Figure 5F, PC1 and PC2 accounted for 51.36 and 14.95% of the variation, respectively, totaling 66.31%. Different colors represent different experimental groups: the FF2 group (orange) had the most tightly clustered samples, indicating a relatively stable microbial structure, while the CON (green) and FF3 (blue) groups were more dispersed, reflecting greater inter-individual variability. However, the PERMANOVA result (*P* = 0.374) revealed no significant differences among groups based on weighted UniFrac distances. This finding suggests that although fermented feed led to compositional shifts in the microbial community, it did not significantly alter the overall abundance-weighted structure. Such a pattern implies that changes were primarily driven by rare or low-abundance taxa, without substantial shifts in dominant microbial populations. Figure 5H shows that within-group distances were smaller, suggesting more similar microbial compositions within groups. The darker color tones between groups indicate that different feed treatments may lead to changes in microbial community composition. However, lighter color overlaps between some groups suggest a certain degree of similarity still exists across treatment groups. Figure 5I reveals that within-group samples generally exhibited lower weighted UniFrac distances, indicating more similar microbial structures within groups. Conversely, higher distances between different groups suggest that feed treatments may cause changes in microbial abundance composition.

### 3.7 Effects of fermented feed on the intestinal microbiota structure in meat ducks

**Figure 6:**
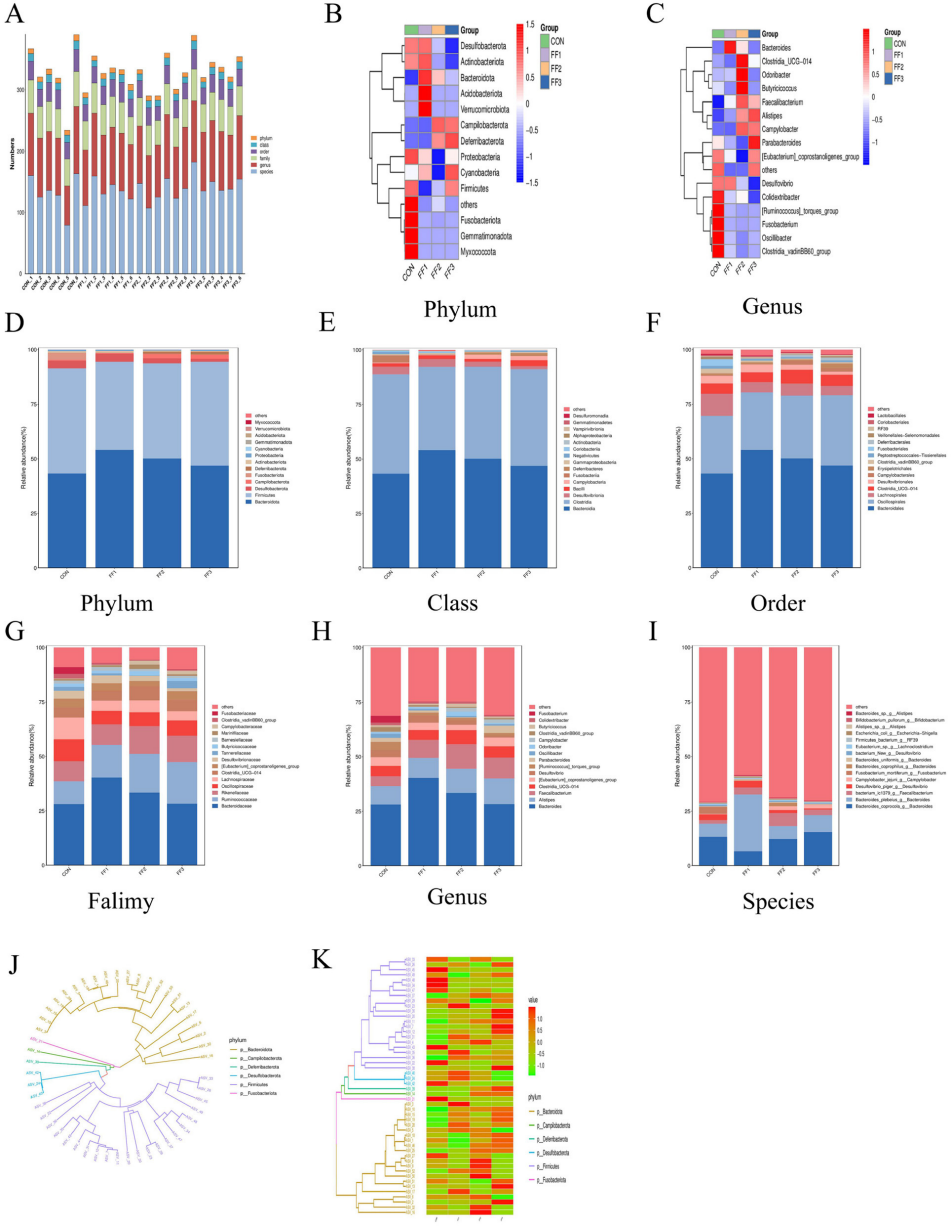
Effects of fermented feed on the intestinal microbiota structure in meat ducks. **(A)** Community-level bar plot. **(B)** Heatmap of the top 15 phyla. **(C)** Heatmap of the top 15 genera. **(D)** Abundance of the top 15 phyla. **(E)** Abundance of the top 15 classes. **(F)** Abundance of the top 15 orders. **(G)** Abundance of the top 15 families. **(H)** Abundance of the top 15 genera. **(I)** Abundance of the top 15 species. **(J)** Phylogenetic tree of the top 50 ASVs. **(K)** Heatmap of the top 50 ASVs. Red indicates higher relative abundance, and blue indicates lower relative abundance.

As shown in Figure 6A, the microbiota was primarily concentrated at the genus and species levels, indicating that microbial richness was mainly reflected at lower taxonomic levels. Differences in abundance at certain taxonomic ranks were observed between the CON group and the fermented feed groups (FF1, FF2, and FF3), especially at the genus and species levels, where some samples in the FF1, FF2, and FF3 groups exhibited higher microbial abundance. This suggests that fermented feed may influence both the composition and abundance of the microbiota. Figures 6B–I show differences in microbial community structures among the CON, FF1, FF2, and FF3 groups. At the phylum level (Figures 6B, D), the dominant phyla were *Bacteroidota* and *Firmicutes*, which together accounted for more than 90% of the total microbiota. Compared to the CON group, the relative abundance of *Bacteroidota* was significantly increased in the FF1, FF2, and FF3 groups (*P* < 0.05), and the abundance of *Deferribacterota* was also significantly increased in the FF2 and FF3 groups. At the genus level (Figures 6C, H), the dominant genera were *Bacteroides, Alistipes, Faecalibacterium, Clostridia_UCG*−*014*, and *[Eubacterium]_coprostanoligenes_group*. Compared to the CON group, the relative abundances of *Bacteroides, Alistipes*, and *Faecalibacterium* were elevated in the FF1, FF2, and FF3 groups, while the abundance of *[Ruminococcus]_torques_group* was reduced.

At the class level (Figure 6E), dominant microbial communities were primarily from *Bacteroidia, Clostridia, Desulfovibrionia*, and *Bacilli*. The relative abundance of *Bacteroidia* increased in the FF1, FF2, and FF3 groups compared to the CON group. At the order level (Figure 6F), dominant groups included *Bacteroidales, Oscillospirales, Lachnospirales, Clostridia_UCG-014*, and *Desulfovibrionales*. *Bacteroidales* showed higher relative abundance in the FF1, FF2, and FF3 groups compared to the CON group. At the family level (Figure 6H), the dominant families were *Bacteroidaceae, Ruminococcaceae, Rikenellaceae, Oscillospiraceae, Lachnospiraceae*, and *Clostridia_UCG-014*. The FF1, FF2, and FF3 groups all showed an increased relative abundance of *Bacteroidaceae, Ruminococcaceae*, and *Rikenellaceae* compared to the CON group. At the species level (Figure 6I), dominant communities included *Bacteroides_coprocola_g__Bacteroides, Bacteroides_plebeius_g__Bacteroides, bacterium_ic1379_g__Faecalibacterium*, and *Desulfovibrio_piger_g__Desulfovibrio*. The FF1, FF2, and FF3 groups had an increased abundance of *bacterium_ic1379_g__Faecalibacterium* compared to the CON group.

Figures 6J, K display the distribution of species from different phyla in the phylogenetic tree. Figure 6J shows that *Bacteroidota* and *Firmicutes* occupied more branches on the tree, indicating greater diversity or divergence within these phyla among the top 50 species. Figure 6K further reveals changes in relative abundance across treatment groups, with notable color differences in certain ASV rows. The FF1, FF2, and FF3 groups showed a higher relative abundance of *Bacteroidota*, while *Firmicutes* and *Desulfobacterota* showed both increases and decreases. The relative abundance of *Campilobacterota* and *Deferribacterota* increased in some samples, whereas *Fusobacteriota* showed a general decrease.

**Figure 7:**
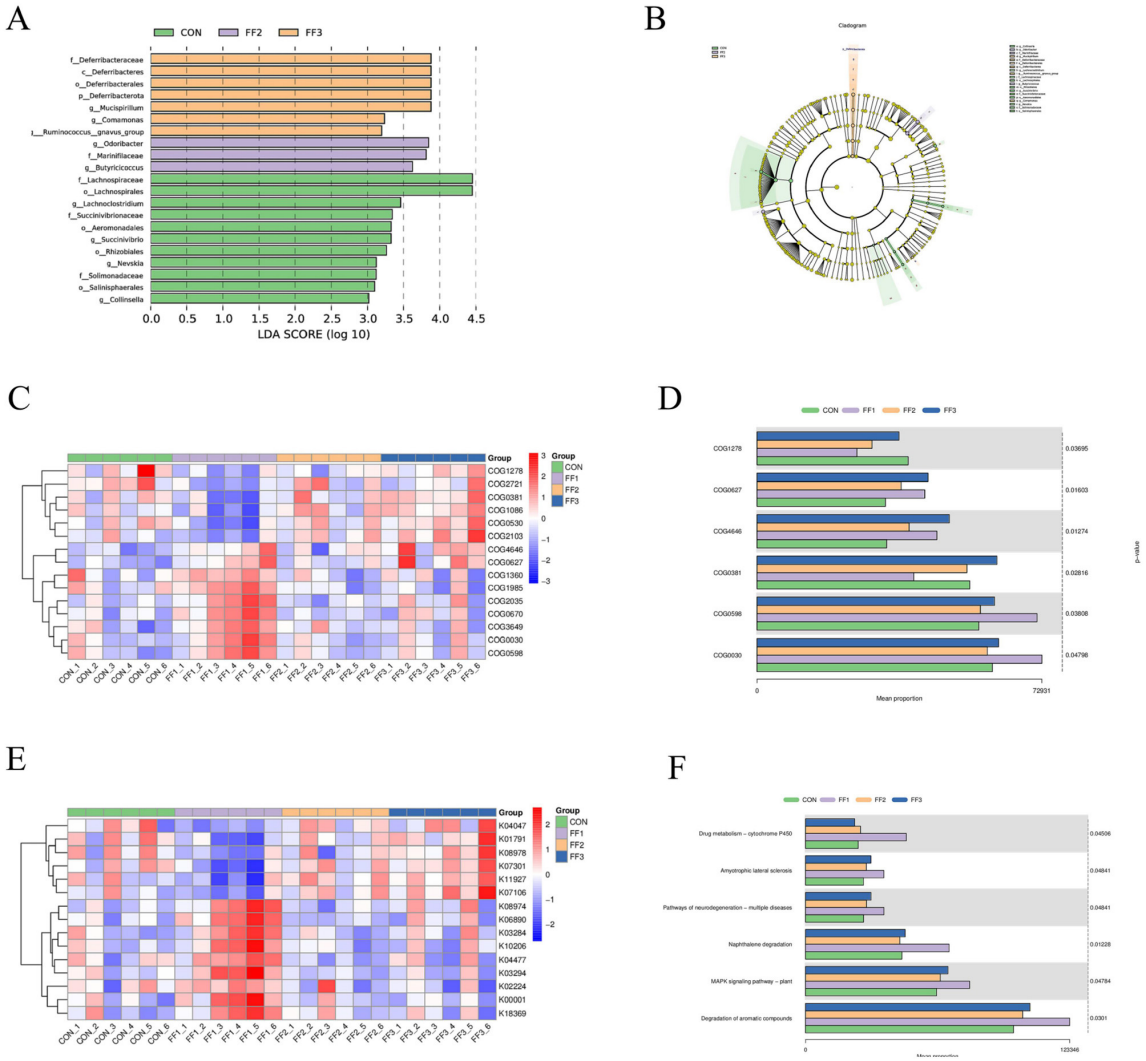
LEfSe analysis/16S-based COG functional prediction/16S-based KEGG functional prediction. **(A)** Differential species LDA score plot **(B)** Cladogram of annotated differential species **(C)** Heatmap of differential COG results **(D)** Bar chart of differential COG results **(E)** Heatmap of differential KEGG results **(F)** Bar chart of differential KEGG results. COG1278: Cold shock protein, CspA family; COG0627: S-formylglutathione hydrolase FrmB; COG4646: Adenine-specific DNA methylase, N12 class; COG0381:UDP-N-acetylglucosamine 2-epimerase; COG0598:Mg2+ and Co2+ transporter CorA; COG0030:16S rRNA A1518 and A1519 N6-dimethyltransferase RsmA/KsgA/DIM1 (may also have DNA glycosylase/AP lyase activity). Red color; a positive correlation, blue color; a negative correlation.

As shown in Figures 7A, B, there were differences in microbial community structures between the CON group and the FF1, FF2, and FF3 groups. A total of 10 potential biomarkers [linear discriminant analysis (LDA) > 3.5] were identified across all groups. In the FF2 group, three potential biomarkers were identified: at the family level: *Marinifilaceae*; at the genus level: *Odoribacter* and *Butyricicoccus*. In the FF3 group, five potential biomarkers were identified: at the phylum level: *Deferribacterota*; at the class level: *Deferribacteres*; at the order level: *Deferribacterales*; at the family level: *Deferribacteraceae*; at the genus level: *Mucispirillum*. These results indicate that fermented feed significantly altered the composition of the microbial community. As shown in Figure 7C, distinct differences were observed in COG functional categories among the CON, FF1, FF2, and FF3 groups. Combined with Figure 7D, the proportions of COG categories COG0627, COG4646H, and COG0598 were higher in the FF1, FF2, and FF3 groups compared to the CON group. In contrast, COG1278, COG0381, and COG0030 were more abundant in the CON group than in the fermented feed treatment groups. Figures 7E, F show that feeding with fermented feed altered the KEGG functions of intestinal microbiota in meat ducks. In the KEGG pathways of amyotrophic lateral sclerosis, pathways of neurodegeneration—multiple diseases, MAPK signaling pathway—plant, and degradation of aromatic compounds, the FF1, FF2, and FF3 groups had higher proportions compared to the CON group.

### 3.8 Correlation analysis

**Figure 8:**
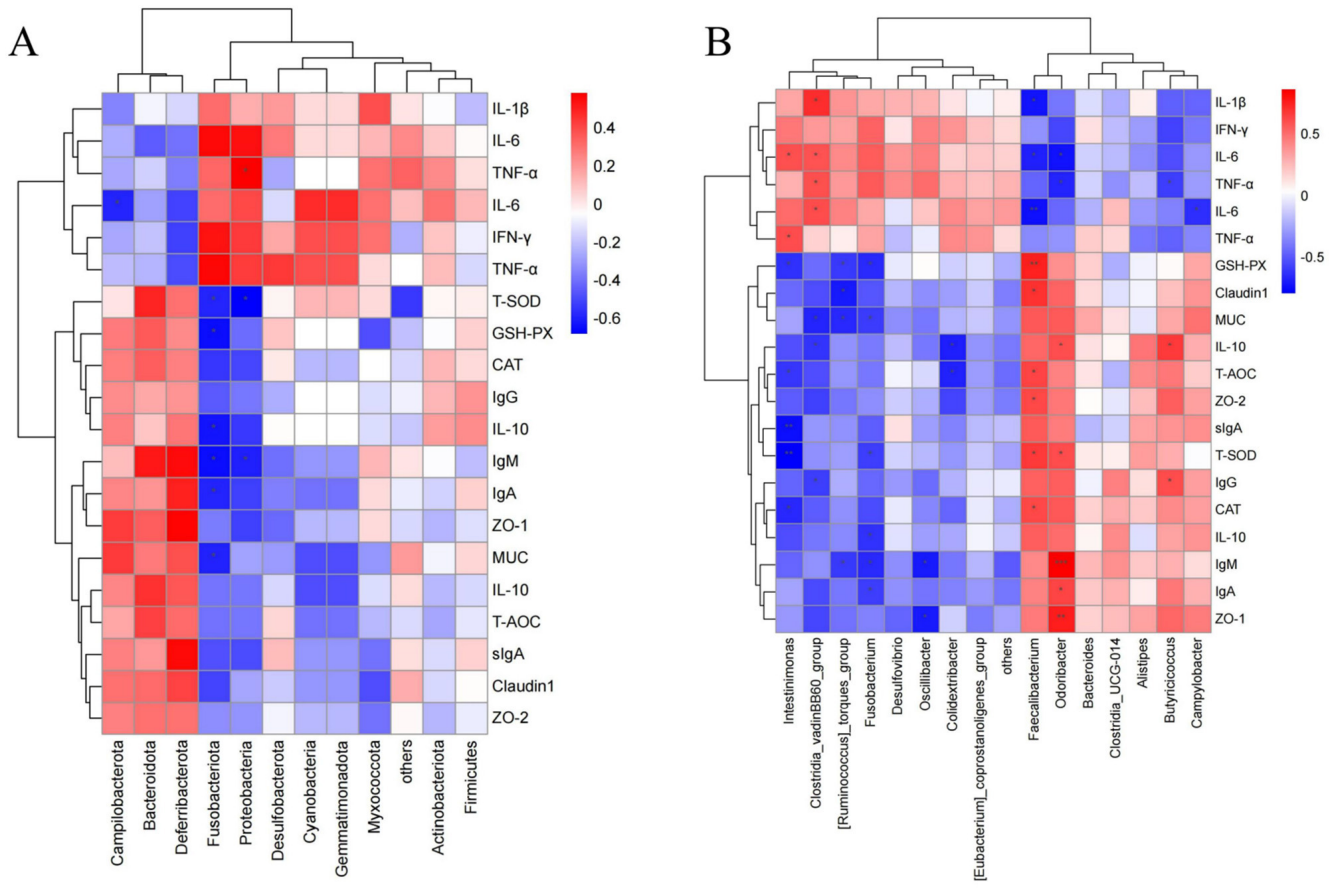
Correlation heatmap **(A)** Correlation between gut microbiota at the phylum level and serum antioxidants, immunoglobulins, and inflammatory factors. **(B)** Correlation between gut microbiota at the genus level and serum antioxidants, immunoglobulins, and inflammatory factors. In the figure, orange-red represents a positive correlation, and blue represents a negative correlation. The deeper the color, the stronger the correlation, while the closer the color is to white, the closer the correlation is to zero. Asterisks in the figure indicate significance: ^***^ represents a correlation *P*-value <0.001, ^**^ represents a correlation *P*-value <0.01, and ^*^ represents a correlation *P*-value <0.05 (indicating significant correlation).

As shown in Figure 8A, at the phylum level, *Proteobacteria* showed a significant positive correlation with TNF-α (*P* < 0.05), while significant negative correlations were observed between GSH-Px and *Fusobacteriota*, T-SOD and *Fusobacteriota*, T-SOD and *Proteobacteria*, IL-6 and *Campilobacterota*, MUC and *Fusobacteriota*, IL-10 and *Fusobacteriota*, IgA and *Fusobacteriota*, IgM and *Fusobacteriota*, and IgM and *Proteobacteria* (*P* < 0.05*)*. Figure 8B shows that at the genus level, significant positive correlations were observed between IL-10 and *Odoribacter*, IL-10 and *Butyricicoccus*, TNF-α and *Clostridia_vadinBB60_group*, T-SOD and *Faecalibacterium*, T-SOD and *Odoribacter*, IL-6 and *Clostridia_vadinBB60_group*, IgG and *Butyricicoccus*, IL-1β and *Clostridia_vadinBB60_group*, CAT and *Faecalibacterium*, ZO-2 and *Faecalibacterium*, Claudin1 and *Faecalibacterium*, IgA and *Odoribacter*, TNF-α and *Intestinimonas*, IL-6 and *Clostridia_vadinBB60_group*, IL-6 and *Intestinimonas*, and T-AOC and *Faecalibacterium* (*P* < 0.05). GSH-Px showed a significant positive correlation with *Faecalibacterium*, and ZO-1 with *Odoribacter* (*P* < 0.01), while IgM showed a significant positive correlation with *Odoribacter* (*P* < 0.001). Significant negative correlations were found between GSH-Px and *[Ruminococcus]_torques_group*, GSH-Px and *Fusobacterium*, GSH-Px and *Intestinimonas*, IL-10 and *Clostridia_vadinBB60_group*, IL-10 and *Colidextribacter*, TNF-α and *Odoribacter*, TNF-α and *Butyricicoccus*, T-SOD and *Fusobacterium*, IL-6 and *Campylobacter*, IgG and *Clostridia_vadinBB60_group*, IL-1β and *Faecalibacterium*, CAT and *Intestinimonas*, MUC and *[Ruminococcus]_torques_group*, MUC and *Fusobacterium*, MUC and *Clostridia_vadinBB60_group*, IL-10 and *Fusobacterium*, Claudin1 and *[Ruminococcus]_torques_group*, ZO-1 and *Oscillibacter*, IgA and *Fusobacterium*, IgM and *[Ruminococcus]_torques_group*, IgM and *Fusobacterium*, IgM and *Oscillibacter*, IL-6 and *Faecalibacterium*, IL-6 and *Odoribacter*, T-AOC and *Colidextribacter*, and T-AOC and *Intestinimonas* (*P* < 0.05). T-SOD was negatively correlated with *Intestinimonas*, IL-6 with *Faecalibacterium*, and sIgA with *Intestinimonas* (*P* < 0.01).

## 4 Discussion

The growth performance indicators of meat ducks are critically important to the duck farming industry. They not only serve as key metrics for evaluating the growth and development of meat ducks and the efficiency of farming operations but also help assess the practical value of fermented feed in duck production. In this study, fermented feed significantly enhanced production performance in Cherry Valley ducks. Both FF1 and FF2 groups showed marked increases in final body weight and average daily gain compared to the control, alongside a notable reduction in feed conversion ratio, indicating improved nutrient utilization. This effect may be associated with probiotic metabolites in the fermented feed, such as organic acids, enzymes, and short-chain fatty acids (23–25), which improve the intestinal environment and facilitate nutrient digestion and absorption. Recent studies have shown that *Bacillus paralicheniformis* strains can produce short-chain fatty acids (SCFAs) suchs as acetate and enhance intestinal barrier integrity, while also modulating inflammatory responses and gut microbiota composition in murine colitis models (24). In addition, genomic analysis of *B. paralicheniformis* BP9 has revealed diverse antimicrobial biosynthetic gene clusters and traits like biofilm formation, swarming mobility, and colonization capacity, suggesting strong antagonistic potential against pathogens and environmental adaptability (26). These functional properties may synergistically contribute to improved gut health and host performance. Building on improvements in growth performance, slaughter performance, and organ indices further supported the nutritional regulatory potential of the fermented feed. In this study, following supplementation with varying levels of fermented feed, the breast muscle yield was significantly higher in the FF1 and FF3 groups. This may be related to the production of beneficial metabolites by *Bacillus paralicheniformis* during the fermentation process, such as small peptides, free amino acids, and antimicrobial peptides (10, 27). These substances not only improve feed digestibility (28) but may also promote protein deposition and enhance amino acid utilization, thereby contributing to muscle development (29, 30).

Serum antioxidant and immune indicators are crucial for meat duck health, as they are key parameters reflecting the health status of the ducks. Specifically, serum IgA levels were elevated across the FF groups, indicating improved mucosal immunity and a heightened defense against pathogens entering through the respiratory and gastrointestinal tracts (31). This enhancement likely contributes to reduced infection risk and better overall health and productivity. In the FF2 group, antioxidant capacity—as reflected by increased T-AOC levels and elevated activities of GSH-Px, T-SOD, and CAT—was markedly improved, accompanied by significant increases in IgG and IgM. Similar trends in antioxidant enzyme activity were observed in the FF3 group, supporting the role of fermented feed in enhancing systemic antioxidant defenses and immune responses. This improvement in antioxidant and immune levels may be closely related to the role of *Bacillus paralicheniformis* during fermentation. As a probiotic spore-forming bacterium (9), *Bacillus paralicheniformis* not only secretes various antioxidant enzymes and metabolically active substances (10, 27), but also colonizes the intestine, activates the immune system, and enhances the secretion of immunoglobulins (27). Additionally, the rich small peptides and probiotic metabolites (10, 27) in fermented feed may also synergistically enhance the antioxidant enzyme activity in the body, reducing oxidative stress (30). These findings are consistent with previous studies indicating that feed-based interventions can significantly improve antioxidant capacity and immune responses through microbiota-mediated mechanisms (32, 33). Regarding inflammatory responses, fermented feed supplementation led to the downregulation of pro-inflammatory cytokines IL-6, TNF-α, and IFN-γ across FF groups, with IL-1β levels also significantly reduced in the FF2 group. Conversely, anti-inflammatory IL-10 expression was significantly increased in FF2 but decreased in FF1, indicating a nuanced, dose-dependent immune modulation. This bidirectional regulation of inflammatory factors may be related to differences in the concentration and composition of microbial fermentation products in the fermented feed. Moderate amounts of fermentation products such as short-chain fatty acids, small peptides, and lactic acid can inhibit the release of pro-inflammatory factors while promoting the expression of anti-inflammatory factors (24). Furthermore, *Bacillus paralicheniformis*, as the main fermentation bacterium, plays an important role in regulating macrophage polarization and T cell activity (34, 35), particularly by reducing the secretion of inflammatory mediators such as IL-6 and TNF-α, while upregulating IL-10 expression, thus suppressing excessive inflammatory responses. These immunomodulatory effects, such as the downregulation of IL-6 and TNF-α and the upregulation of IL-10, are consistent with previous findings in Barbary ducks supplemented with *Bacillus toyonensis* (21), suggesting a potentially conserved anti-inflammatory mechanism among Bacillus species used in poultry.

As a key organ for nutrient absorption and immune regulation, the integrity of intestinal morphology and barrier function is directly related to the health status of the body. The results of this study showed that fermented feed significantly improved villus height and crypt depth in certain intestinal segments of Cherry Valley ducks, with the most prominent effects observed in the FF2 group. Ileal villus height was significantly increased in all FF groups, while the FF2 group also showed notable increases in both jejunal and duodenal villi, indicating a marked enhancement in the structural morphology associated with nutrient absorption throughout the small intestine. However, the duodenal villus height in the FF1 and FF3 groups decreased, which may be related to the concentration, acidity, or viable state of fermentation products affecting the intestinal epithelial cells. This pattern suggests a dose-dependent effect of fermented feed supplementation, where moderate dosing (FF2) optimizes intestinal morphology, but both lower (FF1) and higher (FF3) doses might lead to suboptimal effects. The reduction in villus height in FF1 and FF3 groups could be due to insufficient beneficial metabolites or probiotics in FF1, failing to stimulate intestinal development adequately, while in FF3, excessive fermentation products might cause local mucosal irritation, altered pH, or microbial imbalance that negatively impacts villus integrity. Despite the observed reduction in duodenal villus height in the FF3 group, there were no signs of systemic toxicity or physiological stress. Growth performance, organ indices, and key antioxidant and immune markers remained comparable to those of the control group, suggesting that high-dose fermented feed did not induce adverse health effects. These findings are in line with previous studies reporting non-linear, hormetic effects of probiotics and fermented feed on intestinal morphology (36, 37). Further research, particularly involving histopathological evaluation, is needed to clarify the cellular-level mechanisms underlying these dose-specific responses. Changes in crypt architecture further support the morphological benefits of fermented feed. Ileal crypt depth was significantly increased in all FF groups, while duodenal crypts were significantly shallower in FF1 and FF2. Combined with higher villus height, this indicates an overall optimization of intestinal structure toward improved digestion and absorption. The villus height to crypt depth ratio is also an important indicator of intestinal function, which was significantly increased in the ileum and duodenum of the FF2 group, while significantly decreased in the duodenum of the FF3 group. These changes may be closely related to the metabolites released by *Bacillus paralicheniformis* during the fermentation process, such as small peptides, organic acids, and short-chain fatty acids (24). These active substances can serve as energy sources for intestinal epithelial cells, promote villus growth (38), regulate local pH and microbial communities, and reduce damage to intestinal structures caused by harmful bacteria (39). The synergistic action of probiotics and substrates during fermentation can release various bioactive compounds, further enhancing the integrity and efficiency of the intestinal nutrient absorption structure (38, 40). Beyond structural changes, fermented feed also modulated intestinal barrier gene expression and immune signaling. The relative mRNA levels of *Claudin-1, ZO-1, ZO-*2, and *MUC* were significantly elevated in all FF groups, indicating that fermented feed can effectively enhance the intestinal epithelial barrier function and improve resistance to pathogenic microorganisms (41). Concurrently, pro-inflammatory cytokine *IL-6* was consistently downregulated across all FF groups, while anti-inflammatory *IL-10* was upregulated in FF2 and FF3. *TNF-*α expression decreased in FF1 and FF2, suggesting effective suppression of local intestinal inflammation. Regarding local intestinal immune function, the expression of the *sIgA* gene was significantly upregulated in the FF1 and FF2 groups, suggesting that fermented feed can promote the secretion of mucosal immune factors and enhance the immune defense barrier of the intestine. These results may be due to the fact that *Bacillus paralicheniformis* in the fermented feed can not only produce various beneficial metabolites (10, 27) but also has good colonization ability and intestinal stability (42). Its released immune-regulating factors may enhance the function of the intestinal mucosal immune system by activating local immune signaling pathways (41). Similar improvements in intestinal cytokine profiles and mucosal immunity were also reported in Barbary ducks supplemented with *Bacillus toyonensis*, supporting a potentially conserved mechanism among Bacillus species for gut immune modulation (21).

As an important regulatory factor of host immunity and metabolic function, the structural stability and diversity of the intestinal microbiota have a profound impact on animal health. The results of this study showed that although there were 496 shared ASVs among the four groups, each treatment group also exhibited its own distinct microbial characteristics. Among them, the FF3 group had the highest number of unique ASVs (419), indicating that its microbiota underwent significant changes under the intervention of fermented feed. PERMANOVA analysis based on Unweighted UniFrac distance revealed significant differences in microbial composition among treatment groups (*P* = 0.046), whereas the Weighted UniFrac distance did not show statistical significance (*P* = 0.374). This suggests that the intervention altered the presence or absence of certain low-abundance taxa, rather than the relative abundance of dominant microbial groups. In other words, compositional shifts occurred without substantial changes in the overall abundance-weighted community structure. These changes may be attributed to the rich array of active metabolites in fermented feed, such as short-chain fatty acids, small peptides, and organic acids (10, 24, 27), which can promote colonization and stabilization of beneficial bacteria by regulating intestinal pH, inhibiting pathogenic bacteria, and providing substrates for probiotics (26, 39, 42). *Bacillus paralicheniformis*, as one of the dominant strains, possesses strong heat and acid resistance due to its spore-forming capability (8), allowing it to remain active after passing through the gastrointestinal tract. It can colonize the gut and produce antimicrobial peptides, extracellular enzymes, and other bioactive compounds (23, 43) that suppress harmful bacteria and promote the establishment of intestinal microbial balance (28). Although alpha diversity indices such as Shannon and Chao1 showed no significant differences among the groups, this does not contradict the observed compositional changes in the gut microbiota. Alpha diversity reflects the richness and evenness of species within individual samples and its stability suggests that the overall microbial diversity remained relatively balanced. In contrast, the significant PERMANOVA result based on Unweighted UniFrac distances (*P* = 0.046) and LEfSe analysis revealed distinct differences in community composition among groups. This indicates that fermented feed primarily affected the presence or absence of certain taxa, particularly those with low abundance, without drastically altering the total diversity. Such findings are consistent with previous studies reporting that dietary interventions can reshape microbial composition while maintaining internal diversity homeostasis (44, 45). Further analysis of the microbial community structure at multiple taxonomic levels revealed that fermented feed significantly influenced the composition and abundance of cecal microbiota in meat ducks, especially at the genus and species levels. The microbial quantity in the FF groups was generally higher than that in the CON group, indicating that fermented feed could promote the proliferation of specific beneficial bacteria. At the phylum level, *Bacteroidota* and *Firmicutes* were the dominant phyla in all treatment groups, together accounting for over 90%, and serving as core components in the regulation of gut metabolism and homeostasis. After feeding with fermented feed, the relative abundance of *Bacteroidota* significantly increased in all FF groups, suggesting preferential enrichment under the influence of fermented feed. Additionally, the relative abundance of *Deferribacterota* significantly increased in the FF2 and FF3 groups, which may be related to its functions in energy metabolism and iron reduction (46). At the genus level, *Bacteroides, Alistipes*, and *Faecalibacterium* significantly increased. *Bacteroides* is a typical anaerobic fermentative genus that decomposes complex polysaccharides and produces short-chain fatty acids (SCFAs), playing a positive role in gut barrier function and energy metabolism (47–49); *Alistipes* has anti-inflammatory potential and is believed to alleviate intestinal inflammation in animal models (50); *Faecalibacterium bacterium_ic1379* was upregulated in all FF groups, and its strong butyrate-producing capacity helps enhance the mucosal barrier and improve the intestinal environment (51, 52). At the class, order, and family levels, fermented feed increased the abundance of dominant taxa such as *Bacteroidia* (class), *Bacteroidales* (order), *Bacteroidaceae, Rikenellaceae*, and *Ruminococcaceae* (families). These groups play important roles in maintaining gut microbial balance, promoting fiber degradation, and generating SCFAs (53) and their increased abundance may be due to the nutritional support provided by fermented products such as small peptides and organic acids (54). At the species level, the upregulation of *Faecalibacterium bacterium_ic1379* not only helps regulate inflammatory responses but may also promote mucosal immunity and epithelial repair via SCFAs (55–57). Phylogenetic tree distribution analysis showed that *Bacteroidota* and *Firmicutes* were the most widely distributed among the top 50 species, indicating their core status in the functional construction of the microbiota. Significant color differences in certain ASVs among different treatment groups also suggested that fermented feed, especially in the FF2 and FF3 groups, could significantly affect the abundance of some key species. These changes echo the previously observed enhancements in intestinal barrier function, downregulation of inflammatory cytokines, and improvements in antioxidant capacity, indicating that *Bacillus paralicheniformis* and its functional metabolites produced during fermentation may promote host health by modulating the gut microecology. *Bacillus paralicheniformis* not only possesses strong probiotic properties, such as producing antimicrobial peptides, degrading proteins, and inhibiting pathogenic bacterial colonization (23, 26) but also cooperates with other microorganisms in fermented feed to promote the enrichment and stability of beneficial bacteria, thereby further enhancing intestinal function and host immunity (38, 40).

To further investigate the specific regulatory effects of fermented feed on the intestinal microbiota of meat ducks, representative potential microbial biomarkers in each group were identified through LEfSe analysis (LDA > 3.5). The results showed that, compared with the CON group, the FF2 and FF3 groups enriched three and five microbial biomarkers, respectively, further confirming that fermented feed has a directional reshaping effect on microbiota structure. In the FF2 group, three key biomarkers were identified, including *Marinifilaceae* at the family level and *Odoribacter*, and *Butyricicoccus* at the genus level. *Odoribacter* is closely related to short-chain fatty acid production and has functions in immune regulation and intestinal environment improvement (58); *Butyricicoccus* is an important butyrate-producing bacterium, and butyrate, as a major energy source for epithelial cells, plays multiple roles including anti-inflammation and promoting mucosal repair (59, 60). The upregulation of these beneficial bacteria in the FF2 group may be one of the reasons for its notable performance in immune regulation and barrier enhancement. In the FF3 group, five biomarkers were significantly enriched, all related to the phylum *Deferribacterota*, including the phylum, class, order, family, and genus levels, and were ultimately identified as belonging to the genus *Mucispirillum*. *Mucispirillum* typically colonizes the mucus layer of the intestine and is associated with host mucosal immunity (61). Its enrichment in the FF3 group may be related to mucosal barrier regulation and mucus metabolism, further indicating the potential of FF3 feed in establishing intestinal microbial homeostasis. Additionally, functional prediction analysis showed that fermented feed not only altered microbial structure but also had significant effects on microbial functional potential. Although the FF3 group showed substantial changes in microbiota composition and some favorable microbial biomarkers, its overall physiological and immune performance was less consistent compared to the FF2 group. This discrepancy may stem from the higher inclusion level of fermented feed in FF3, which could have introduced a threshold effect or dose-dependent response. At high concentrations, certain fermentation by-products or metabolic acids might exceed optimal physiological tolerance, potentially leading to mild gastrointestinal stress or microbiota imbalance in some individuals. Moreover, high levels of fermentation compounds may affect feed palatability, reducing intake and nutritional consistency. The large number of unique ASVs and strong compositional shifts in the FF3 group further suggest that microbial modulation may have exceeded a stable functional threshold, resulting in individual variability. Future studies should explore the dose–response relationship of fermented feed inclusion to optimize beneficial outcomes while minimizing variability or unintended effects. In the COG functional categories, the FF1, FF2, and FF3 groups had significantly higher proportions in categories COG0627, COG4646H, and COG0598 compared to the CON group. These functions are mostly related to material metabolism, energy conversion, and cellular process regulation (62, 63), indicating that fermented feed may enhance nutrient absorption and energy utilization by boosting microbial metabolic potential. In the KEGG pathway analysis, the FF treatment groups showed significantly increased enrichment in pathways such as amyotrophic lateral sclerosis, pathways of neurodegeneration–multiple diseases, MAPK signaling pathway–plant, and degradation of aromatic compounds. These pathways typically represent microbial responses to host environmental stress, signal regulation, and degradation of exogenous organics, which may reflect improved adaptability, enhanced environmental sensing, and more diverse and efficient metabolic activity of the intestinal microbiota in ducks fed with fermented feed. These functional shifts are likely driven by the metabolic activity of *Bacillus paralicheniformis*, which produces a wide array of bioactive small molecules during fermentation, including organic acids, antimicrobial peptides, and extracellular enzymes. These compounds can directly modify the feed matrix to improve its bioavailability (10, 26), while also enriching beneficial bacterial populations, suppressing pathogens, and modulating host-microbiota metabolic interactions (26, 64, 65). Consistent with our findings, Pan et al. (32) reported that dietary phosphorus levels in Hu lambs significantly altered serum biochemical indices, amino acid metabolism pathways, and microbial fermentation parameters, which were closely associated with improved growth performance and microbial protein synthesis. Their study highlights the importance of nutritional modulation of microbial communities and metabolic profiles in optimizing animal health and performance, which supports the role of fermented feed in promoting beneficial microbial activity and metabolic output in our study.

In the correlation analysis between microbes and host indicators, the results showed that some key phyla and genera were closely related to intestinal barrier function, inflammatory factors, and antioxidant capacity, further suggesting that fermented feed may improve host health by modulating these microbial populations. *Bacillus paralicheniformis*, as a key functional strain in fermented feed, may participate in this process through its metabolic products. For example, Proteobacteria were positively correlated with the pro-inflammatory factor TNF-α and negatively correlated with the antioxidant enzyme T-SOD and immunoglobulin IgM, suggesting that the increase in this phylum may be associated with inflammation and oxidative stress. In contrast, probiotic genera such as *Faecalibacterium, Odoribacter*, and *Butyricicoccus* showed significant positive correlations with anti-inflammatory or immune-protective factors like IL-10, T-SOD, IgA, and IgG. These results indicate that fermented feed not only helps enrich beneficial bacteria and improve intestinal integrity but may also modulate host immunity and oxidative balance. One possible mechanism involves microbial-derived short-chain fatty acids (SCFAs), such as butyrate, which are known to influence immune and epithelial function through signaling pathways like GPR41/43 and histone deacetylase (HDAC) inhibition (56, 66–68). Thus, the modulation of gut microbiota by *Bacillus paralicheniformis* LN33-fermented feed could potentially act through SCFA-mediated signaling to regulate host physiological responses.

Despite the promising findings, this study has several limitations. First, it was conducted under controlled experimental conditions, which may not fully replicate commercial production environments with variable stressors and pathogen exposure. Second, only a single probiotic strain (*Bacillus paralicheniformis* LN33) and three inclusion levels were tested; the potential effects of other strains or fermentation conditions were not explored. Third, the study duration was limited to 28 days, which may not capture long-term impacts on growth performance or gut microbiota stability. Fourth, although 16S rRNA gene sequencing revealed significant shifts in microbial composition and KEGG/COG functional predictions were included, no direct functional validation such as metabolomic profiling or short-chain fatty acid (SCFA) quantification was performed. Without these, it remains unclear whether the predicted pathways are enzymatically active or physiologically relevant, as gene expression may be influenced by substrate availability or regulatory constraints. Fifth, the study did not include a heat-inactivated or sterilized fermented feed control group, making it difficult to distinguish whether the observed benefits were due to live *B. paralicheniformis* or its fermentation-derived metabolites. This limits the ability to clarify whether effects were driven by probiotic activity or postbiotic mechanisms. Additionally, while robust correlations were observed between microbial taxa and host physiological parameters (immune, antioxidant, and intestinal barrier functions), causality has not been established, and mechanistic links remain speculative.

Therefore, future studies should extend feeding trials over longer durations, explore other probiotic strains or combinations, and incorporate mechanistic approaches such as metabolomics, SCFA quantification, gnotobiotic animal models, inclusion of inactivated microbial controls, and transcriptomic analyses to validate and deepen understanding of the microbiome-host interactions and their impact on health and performance.

## 5 Conclusions

**Figure 9:**
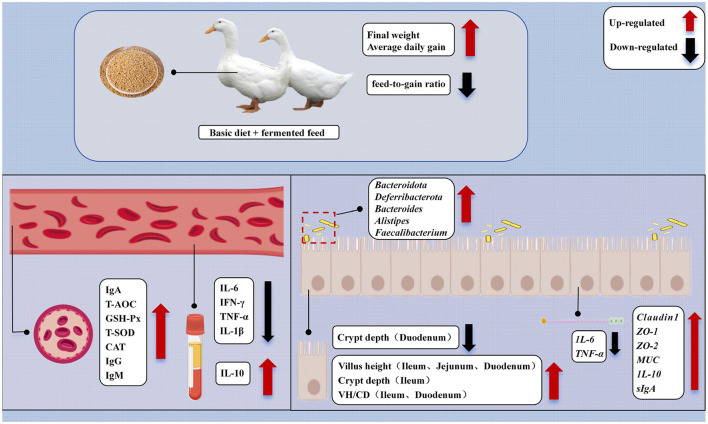
Mechanisms of the impact of dietary *Bacillus paralicheniformis* LN33 fermented feed on the health of Cherry Valley ducks.

This study systematically evaluated the comprehensive effects of *Bacillus paralicheniformis* LN33-fermented feed on growth performance, intestinal morphology, immune status, antioxidant capacity, barrier function, and gut microbiota composition in meat ducks. The results demonstrated that *Bacillus paralicheniformis* LN33-fermented feed significantly increased final body weight and average daily gain while reducing the feed conversion ratio. It enhanced the activities of key antioxidant enzymes such as GSH-Px, T-SOD, and CAT, thereby strengthening the body's antioxidant defense system. The fermented feed also significantly improved the expression of tight junction proteins, thereby enhancing intestinal barrier function. At the same time, it reduced levels of pro-inflammatory cytokines and increased the expression of anti-inflammatory factors. Gut microbiota analysis further revealed that the feed promoted the enrichment of beneficial bacteria such as *Faecalibacterium, Odoribacter*, and *Butyricicoccus*, contributing to the maintenance of microbial homeostasis. Correlation analysis revealed that these key genera were significantly associated either positively or negatively with immune, antioxidant, and intestinal barrier indicators, further supporting the functional relevance of the gut microbiota (Figure 9). In conclusion, moderate supplementation with *Bacillus paralicheniformis* LN33-fermented feed, especially at the 3% inclusion level (FF2), was the most effective in synergistically enhancing growth performance, improving intestinal health, and regulating immune and oxidative status in meat ducks, likely through optimizing gut microbial structure.

## Data Availability

The original contributions presented in the study are publicly available. This data can be found here: https://www.ncbi.nlm.nih.gov/bioproject/PRJNA1289083.
